# Comparison of cold crystalloid and colloid infusions for induction of therapeutic hypothermia in a porcine model of cardiac arrest

**DOI:** 10.1186/cc13068

**Published:** 2013-10-16

**Authors:** Roman Skulec, Anatolij Truhlar, Zdenek Turek, Renata Parizkova, Pavel Dostal, Shawn Hicks, Christian Lehmann, Vladimir Cerny

**Affiliations:** 1 Department of Anesthesiology and Intensive Care, Charles University in Prague, Faculty of Medicine in Hradec Kralove, University Hospital Hradec Kralove, Sokolská 581, Hradec, Kralove 500 05, Czech Republic; 2 Emergency Medical Service of the Central Bohemian Region, Prof. Veseleho 461, Beroun 266 01, Czech Republic; 3 Hradec Kralove Region Emergency Medical Services, Hradecka 1690/2A, Hradec Kralove 500 12, Czech Republic; 4 Department of Anesthesia, Pain Management and Perioperative Medicine, Dalhousie University, 1276 South Park St., Halifax NS B3H 2Y9, Canada

## Abstract

**Introduction:**

Large-volume cold intravenous infusion of crystalloids has been used for induction of therapeutic hypothermia after cardiac arrest. However, the effectiveness of cold colloids has not been evaluated. Therefore, we performed an experimental study to investigate the cooling effect of cold normal saline compared to colloid solution in a porcine model of ventricular fibrillation.

**Methods:**

Ventricular fibrillation was induced for 15 minutes in 22 anesthetized domestic pigs. After spontaneous circulation was restored, the animals were randomized to receive either 45 ml/kg of 1°C cold normal saline (Group A, 9 animals); or 45 ml/kg of 1°C cold colloid solution (Voluven®, 6% hydroxyethyl starch 130/0.4 in 0.9% NaCl) during 20 minutes (Group B, 9 animals); or to undergo no cooling intervention (Group C, 4 animals). Then, the animals were observed for 90 minutes. Cerebral, rectal, intramuscular, pulmonary artery, and subcutaneous fat body temperatures (BT) were recorded. In the mechanical ex-vivo sub study we added a same amount of cold normal saline or colloid into the bath of normal saline and calculated the area under the curve (AUC) for induced temperature changes.

**Results:**

Animals treated with cold fluids achieved a significant decrease of BT at all measurement sites, whereas there was a consistent significant spontaneous increase in group C. At the time of completion of infusion, greater decrease in pulmonary artery BT and cerebral BT in group A compared to group B was detected (−2.1 ± 0.3 vs. -1.6 ± 0.2°C, and −1.7 ± 0.4 vs. -1.1 ± 0.3°C, p < 0.05, respectively). AUC analysis of the decrease of cerebral BT revealed a more vigorous cooling effect in group A compared to group B (−91 ± 22 vs. -68 ± 23°C/min, p = 0.046). In the mechanical sub study, AUC analysis of the induced temperature decrease of cooled solution revealed that addition of normal saline led to more intense cooling than colloid solution (−7155 ± 647 vs. -5733 ± 636°C/min, p = 0.008).

**Conclusions:**

Intravenous infusion of cold normal saline resulted in more intense decrease of cerebral and pulmonary artery BT than colloid infusion in this porcine model of cardiac arrest. This difference is at least partially related to the various specific heat capacities of the coolants.

## Introduction

The technique of large-volume cold intravenous infusion has been found to be a simple, efficient and safe method of inducing therapeutic hypothermia (TH) in cardiac arrest survivors [1,2], therefore, it has been widely adopted [1,3]. However, in many patients, it fails to achieve target body temperature of 34°C [1,4-9]. Several conditions may have an impact on cooling efficiency, indicating that there is room for optimization of the cooling protocol [10]. One of the potential approaches to improve cooling efficacy is to search for the most efficient coolant. Different solutions may exert different cooling effects based on their particular specific heat capacity and their impact on hemodynamics and heat exchange in the body. However, there is a lack of the knowledge on this specific topic. Therefore, we have carried out an experimental study to investigate the cooling effect of rapid intravenous infusion of cold normal saline compared to isotonic iso-oncotic colloid solution in a porcine model of ventricular fibrillation (VF). We hypothesized that equivalent volumes of those types of fluids may cause a different decrease in body temperature (BT).

## Materials and methods

We performed a prospective randomized controlled experimental study on 22 healthy female domestic pigs *(Sus scrofa f. domestica)* weighing 30 to 35 kg (mean 33 ± SD 2 kg). The experiment was performed in the Animal Research Laboratory of the University of Defence, Faculty of Military Health Sciences, Czech Republic. The study protocol was approved by the Animal Investigation Committee of the University of Defence Brno, Faculty of Military Health Sciences Hradec Kralove, Czech Republic and the Departmental Commission for the Protection of Animals of the Ministry of Defence, Prague, Czech Republic. All experimental animals received humane care in compliance with the institutional guidelines. Animals were fasted overnight, but had free access to water. Anesthesia was used in all surgical interventions. We also performed a mechanical substudy without any involvement of humans or experimental animals in the setting of air-conditioned laboratory conditions.

### Animal preparation

The animals were premedicated by intramuscular injection of azaperone (2.0 mg/kg), atropine (0.2 mg/kg) and ketamine (20.0 mg/kg) 30 minutes before surgery. This was followed by ear-vein injection of thiopental (3 mg/kg). The pigs were fixed in the right lateral position on the backboard of the resuscitation system AutoPulse Model 100 (Zoll, Medical Corp. Chelmsford, MA, USA), intubated and mechanically ventilated (Siemens-Elema SV 900C, AB, Solna, Sweden) at 19 breaths/minute, and inspired oxygen fraction (FiO_2_) of 0.4. Tidal volumes were adjusted to maintain end-tidal CO_2_ at 35 to 45 mm Hg. Anesthesia and muscle paralysis were maintained with a continuous infusion of midazolam (0.3 mg/kg/h), fentanyl (5 to 20 μg/kg/h) and pancuronium (0.5 mg/kg/h). In addition to interventions in groups A and B, all animals were continuously given normal saline (B. Braun, Melsungen, Germany) at room temperature (50 ml/h). Vital signs were continuously monitored with two Datex-Ohmeda Type S/5 monitors (Datex-Ohmeda Instrumentarium Corp., Helsinki, Finland) and a Zoll M Series defibrillator (Zoll Medical Corp.). The distinct infusion rate of coolants was controlled by a Power Infuser (Zoll Medical Corp.).

### Measurements

After induction of anesthesia, the thoracic aorta was cannulated via the carotid artery with a 7F 200-mm catheter Certofix Duo (B. Braun) for monitoring of aortic blood pressure. An 8.5F percutaneous sheath introducer Intro-Flex (Edwards Lifesciences LLC, Irvine, CA, USA) was inserted via the internal jugular vein into the superior vena cava to enable insertion of a CCOmbo Volumetrics Pulmonary Artery Catheter (Edwards Lifesciences LLC) for continual monitoring of pulmonary artery BT, right atrial pressure, pulmonary artery pressure, cardiac index, mixed venous oxygen saturation and intermittent measurement of pulmonary artery wedge pressure. A 5-mm diameter burr-hole craniotomy at the upper part of the frontal bone was created on the left side to insert an intracranial pressure monitoring device. A parenchymal probe Codman MicroSensor ICP Transducer (Codman, Johnson & Johnson, Raynham, MA, USA) was inserted 20 mm into the frontal lobe. Cerebral, rectal, intramuscular, and subcutaneous fat BT were continuously recorded using GMH 3250 digital thermometers (Greisinger Electronic, Regenstauf, Germany). A cerebral probe was placed 20 mm into the right hemisphere, on the opposite site of the head from the intracranial pressure probe. Intramuscular and subcutaneous fat BT probes were inserted into the right gluteal muscle and abdominal subcutaneous adipose tissue, respectively. Coronary perfusion pressure (CoPP) was defined as the pressure difference between diastolic aortic pressure and right atrial pressure during the decompression phase. Cerebral perfusion pressure (CPP) was calculated from mean aortic pressure (MAP) and intracranial (ICP) or central venous pressure (CVP) according to the formula:

$$\begin{array}{l} \mathrm{CPP}=\text{MAP}–\text{ICP}\;\mathrm{if}\;\text{ICP}\geq \text{CVP}\;\mathrm{or}\\ \;\text{CPP}=\text{MAP}–\text{CVP}\;\mathrm{if}\;\text{CVP}>\text{ICP}. \end{array}$$

### Experimental protocol

**Figure 1:**
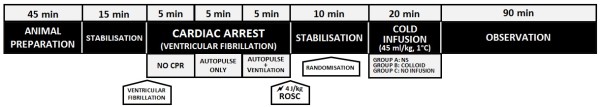
**Flow chart of the animal experimental protocol.** CPR, cardiopulmonary resuscitation; ROSC, return of spontaneous circulation; NS, normal saline.

Figure 1 outlines the protocol flow chart. After animal preparation and stabilization, ventricular fibrillation (VF) was induced with an alternating current of 5 to 10 V using an intra-cardiac bipolar pacing lead introduced into the right ventricle. Cardiac arrest was confirmed as the time point at which aortic pressures dropped down to equal values and the electrocardiogram (ECG) showed VF. The animals were left in non-resuscitated cardiac arrest for 5 minutes. Thereafter, mechanical chest compressions at a rate of 100/minute without ventilation were performed for 5 minutes using the AutoPulse. Mechanical ventilation (10 breaths/minute, FiO_2_ = 1.0, tidal volume 8 ml/kg) was then added. Fifteen minutes after induction of cardiac arrest, a defibrillation shock of 150 J was delivered to restore spontaneous circulation, and repeated if necessary. When return of spontaneous circulation (ROSC) was reached, chest compressions were terminated and ventilatory support was set to the baseline regimen. Thereafter, the pigs were randomly assigned into three groups to receive either 45 ml/kg of 1°C cold normal saline during 20 minutes (Group A, nine animals); or 45 ml/kg of 1°C cold colloid solution (Voluven®, 6% hydroxyethyl starch 130/0.4 in normal saline, Fresenius Kabi AG, Bad Homburg, Germany) during 20 minutes (Group B, nine animals); or to serve as a control group with no cooling intervention (Group C, four animals). During the subsequent 90 minutes, the animals were observed to assess the effect of interventions on BT and hemodynamics. After this period, the animals were sacrificed in deep anesthesia by re-induction of VF, and autopsied.

The aim of the mechanical substudy was to compare the specific heat capacities (SHC) of the coolants used in groups A and B. Normal saline (500 ml) was prepared in a beaker to serve as the experimental cooled solution. After we achieved temperature equilibrium, we poured into the solution 50 ml of 1°C cold normal saline or 50 ml of 1°C cold colloid solution in random order. We proceeded with continuous temperature monitoring of the cooled solution for 120 minutes. Afterwards, we calculated the area under the curve (AUC) for temperature changes. For each coolant, five sessions were repeated and averaged for further analysis.

### Statistical analysis

For the statistical analysis, measurements were taken at the baseline, immediately before cold infusion (10 minutes after ROSC), 10 minutes after the start of cold infusion, after finishing cold infusion, and every 10 minutes during the period of observation until the end of the animal experimental protocol. At every defined time point, we calculated the difference between the actual BT and the BT immediately before cold infusion, for comparison between the groups. In the mechanical substudy, we sampled the temperature of the cooled solution every 20 seconds during 120 minutes of monitoring. For every time point we calculated the difference between the baseline and actual temperature. The AUC from the differences was calculated by a trapezoid rule. Mean values ± SD or percentages were calculated as necessary. Differences between groups were compared using the *χ*^2^ test, and statistical significance was calculated by the Fischer exact test for alternative variables. Statistical significance for continuous variables was determined by the paired Student *t*-test. Data were analyzed using Microsoft Excel 2007 and JMP 3.2 statistical software. A *P*-value of <0.05 was considered statistically significant.

## Results

**Figure 2:**
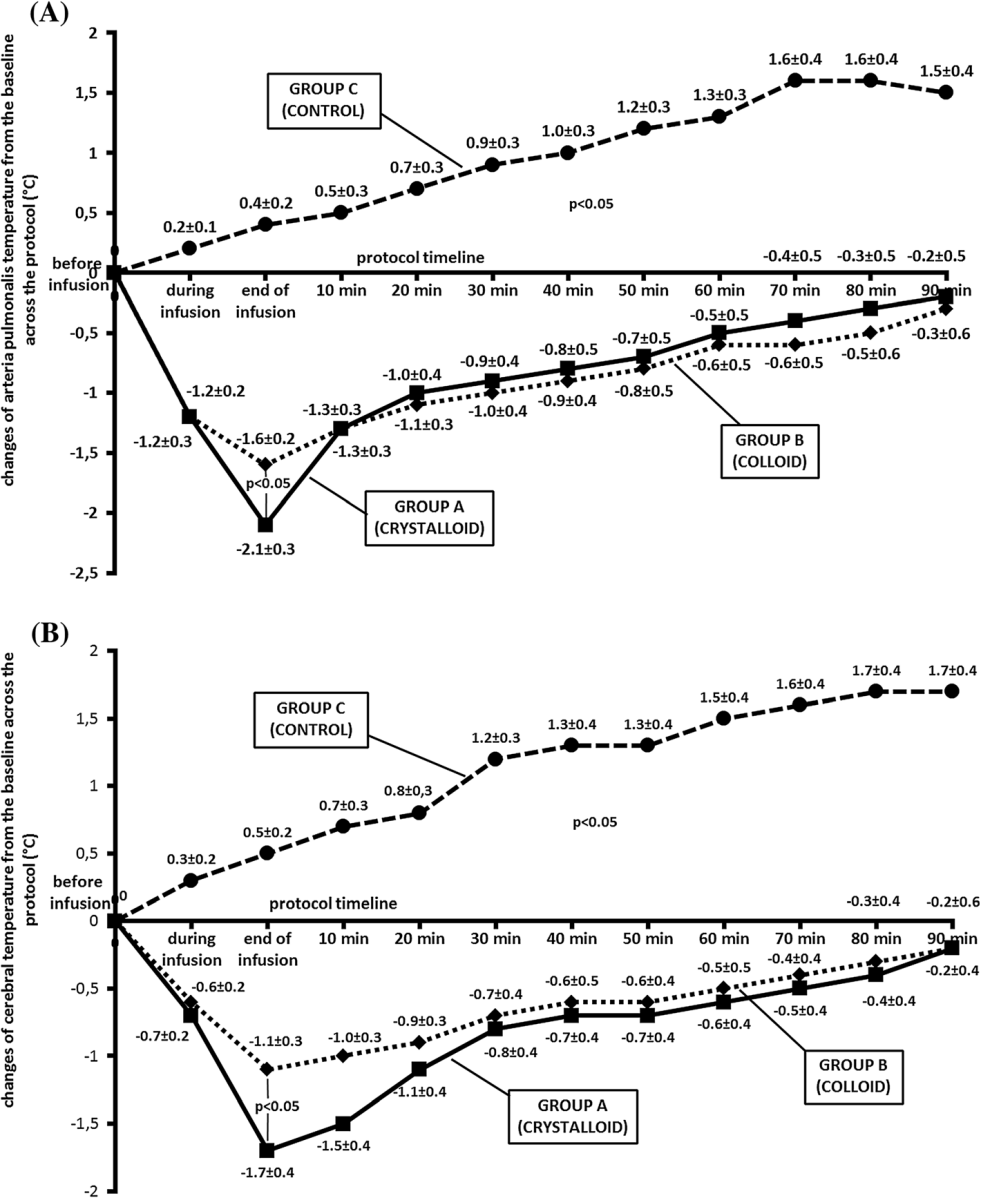
**Course of changes in arteria pulmonalis and cerebral body temperature during the protocol in all groups. (A)** Arteria pulmonalis body temperature (BT). **(B)** Cerebral BT. Solid line, group A; dotted line, group B; dashed line, group C.

There were no significant differences in any physiological variables between the baseline values and values immediately before cold infusion among the three experimental groups (Table 1). The groups were comparable in the number of defibrillation shocks delivered (group A, 1.7 ± 1.0; group B, 1.7 ± 0.7; group C, 1.5 ± 0.6; *P* = 0.936) and in time from induction of VF to ROSC (group A, 16.3 ± 2.0; group B, 16.3 ± 1.4; group C, 16.0 ± 1.1; *P* = 0.936). The animals treated with cold fluids achieved a significant decrease in BT at all measurement sites, whereas there was a consistent spontaneous increase in group C. The differences between the change in BT in group C and groups A or B were significant at all sites at all phases of the protocol, both during the cooling and during the 90 minutes of further monitoring. (Figure 2, Table 2). At the time of completion of the infusion, a significantly greater decrease in pulmonary artery BT and cerebral BT in group A compared to group B was detected (−2.1 ± 0.3 versus −1.6 ± 0.2°C, and −1.7 ± 0.4 versus −1.1 ± 0.3°C, *P* <0.05 respectively) (Figure 2). We did not observe any significant differences in the changes in rectal, intramuscular and subcutaneous fat BT between groups A and B (Table 2). Maximal decrease in BT was recorded at all sites of measurement on completion of the infusion or 10 minutes after finishing the infusion in groups A and B. Although considerable and ongoing re-warming was observed at the pulmonary artery, cerebral, rectal and intramuscular BT measurement sites soon after finishing the infusion until the end of the protocol, a more intense and persistent decrease in the temperature of subcutaneous fat was revealed at these compared to other sites in groups A and B from 20 minutes after the finishing infusion (Table 2).

**Table 1:** Selected values of physiological variables immediately before cold infusion

	**Group A (normal saline)**	**Group B (colloid)**	**Group C (control group)**
Body weight (kg)	-	-	-
BSA (m^2^)	-	-	-
Pulmonary artery BT (mm Hg)	36.7 ± 0.4	36.6 ± 0.9	36.7 ± 0.9
Cerebral BT (°C)	36.8 ± 0.5	36.8 ± 0.8	36.9 ± 1.0
Rectal BT (°C)	36.6 ± 0.4	36.8 ± 0.9	36.7 ± 0.4
Intramuscular BT (°C)	36.7 ± 0.4	36.8 ± 0.9	36.7 ± 1.0
Subcutaneous fat BT (°C)	36.4 ± 0.5	36.6 ± 1.0	36.6 ± 0.9
Cardiac index (l/minute/m^2^)	7.8 ± 1.4	7.4 ± 1.5	7.6 ± 0.5
Intracranial pressure (mm Hg)	11 ± 7	12 ± 6	11 ± 1
Coronary perfusion pressure (mm Hg)	55 ± 20	56 ± 16	56 ± 16
Cerebral perfusion pressure (mm Hg)	62 ± 18	63 ± 17	63 ± 19

*P* >0.05 for all differences between the groups. BSA, body surface area; BT, body temperature.

**Table 2:** Time course of rectal, intramuscular and subcutaneous fat body temperature changes from before infusion until the end of the protocol

**Time point**	**Rectal BT**			**Intramuscular BT**			**Subcutaneous fat BT**		
	**Group A**	**Group B**	**Group C**	**Group A**	**Group B**	**Group C**	**Group A**	**Group B**	**Group C**
**During infusion**	−0.4 ± 0.2	−0.4 ± 0.3	0.2 ± 0.1^a^	−0.6 ± 0.2	−0.5 ± 0.2	0.2 ± 0.2^a^	−0.7 ± 0.4	−0.5 ± 0.2	0.3 ± 0.1^a^
**End of infusion**	−1.2 ± 0.4	−1.1 ± 0.4	0.4 ± 0.2^a^	−1.5 ± 0.3	−1.2 ± 0.3	0.3 ± 0.1^a^	−1.6 ± 0.5	−1.3 ± 0.3	0.5 ± 0.2^a^
**10 minutes**	−1.4 ± 0.3	−1.2 ± 0.3	0.6 ± 0.2^a^	−1.4 ± 0.4	−1.2 ± 0.3	0.4 ± 0.3^a^	−1.7 ± 0.5	−1.5 ± 0.3	0.6 ± 0.2^a^
**20 minutes**	−1.3 ± 0.3	−1.2 ± 0.3	0.7 ± 0.2^a^	−1.2 ± 0.4	−1.1 ± 0.4	0.6 ± 0.3^a^	−1.6 ± 0.5	−1.6 ± 0.3^c^	0.8 ± 0.2^a^
**30 minutes**	−1.1 ± 0.4	−1.1 ± 0.4	0.9 ± 0.2^a^	−1.0 ± 0.4	−1.0 ± 0.4	0.7 ± 0.4^a^	−1.4 ± 0.4	−1.6 ± 0.4^c^	1.0 ± 0.4^a^
**40 minutes**	−1.0 ± 0.4	−1.0 ± 0.4	1.0 ± 0.3^a^	−0.9 ± 0.4	−0.9 ± 0.5	1.1 ± 0.2^a^	−1.3 ± 0.5	−1.6 ± 0.4^c^	1.2 ± 0.3^a^
**50 minutes**	−0.9 ± 0.4	−0.9 ± 0.4	1.1 ± 0.3^a^	−0.8 ± 0.4	−0.8 ± 0.5	1.1 ± 0.5^a^	−1.2 ± 0.4	−1.4 ± 0.5^c^	1.4 ± 0.4^a^
**60 minutes**	−0.9 ± 0.4	−0.9 ± 0.5	1.3 ± 0.3^a^	−0.7 ± 0.4	−0.7 ± 0.6	1.2 ± 0.6^a^	−1.1 ± 0.4	−1.5 ± 0.6^c^	1.6 ± 0.5^a^
**70 minutes**	−0.7 ± 0.4	−0.7 ± 0.6	1.4 ± 0.3^a^	−0.6 ± 0.4	−0.6 ± 0.6	1.3 ± 0.6^a^	−1.1 ± 0.4^b^	−1.4 ± 0.6^c^	1.7 ± 0.5^a^
**80 minutes**	−0.6 ± 0.5	−0.6 ± 0.6	1.5 ± 0.3^a^	−0.5 ± 0.4	−0.5 ± 0.7	1.4 ± 0.7^a^	−1.0 ± 0.4^b^	−1.3 ± 0.6^c^	1.8 ± 0.4^a^
**90 minutes**	−0.6 ± 0.5	−0.6 ± 0.6	1.4 ± 0.3^a^	−0.6 ± 0.4	−0.4 ± 0.8	1.4 ± 0.6^a^	−1.0 ± 0.4^b^	−1.2 ± 0.6^c^	1.8 ± 0.4^a^

Results are presented as mean ± SD. ^a^Significant difference between group C and groups A and B at each site of measurement; ^b^significant difference between subcutaneous fat body temperature (BT) changes in group A and group B temperature changes at all other measurement sites; ^c^significant difference between subcutaneous fat BT changes in group B and group B temperature changes at all other measurement sites.

AUC analysis of the decrease in cerebral BT revealed a more vigorous cooling effect in group A compared to group B (−91 ± 22 versus −68 ± 23°C/minute, *P* = 0.046).

**Figure 3:**
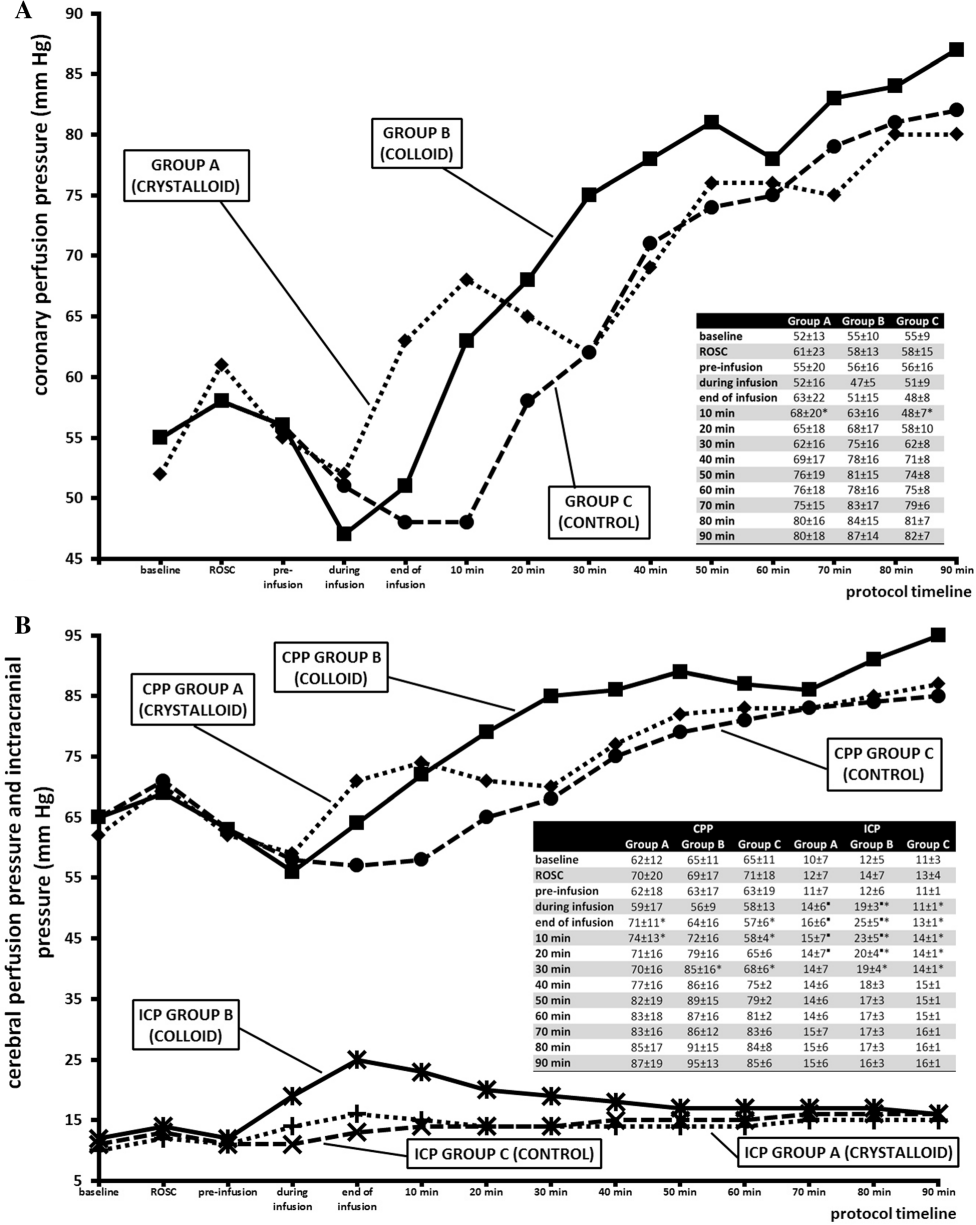
**Values of coronary perfusion pressure (CoPP), cerebral perfusion pressure (CPP) and intracranial pressure (ICP) during the protocol. (A)** CoPP values. **(B)** CPP and ICP values. Asteriks indicate significant differences between group C and group A and/or B. Solid line, group A; dotted line, group B; dashed line, group C. Solid square symbols indicate significant differences between group A and group B. ROSC, return of spontaneous circulation.

Figure 3 demonstrates the hemodynamic effect of interventions in groups A and B. For group C animals, Figure 2 shows a spontaneous temporary decrease in CPP and CoPP followed by progressive increase above baseline values. Rapid infusion of cold normal saline in group A significantly precipitated CoPP restoration and the same trend was identified in group B. The impact of cold infusion on CPP was found similar to that on CoPP. However, in the group B animals, infusion of colloid solution led to a vigorous increase in ICP during the infusion and up to 30 minutes after its administration, whereas the ICP profile in group A traced the group C pattern (Figure 3). The cardiac index continuously decreased in group C until the end of the protocol. Cold infusion in both interventional groups induced a significant increase in the cardiac index, more intense in group B than A, with the maximum in the thirtieth minute after finishing the infusion (group A, 9.0 ± 1.4; group B, 10.4 ± 2.1; group C, 7.0 ± 0.2; *P* <0.05). This was followed by a subsequent decrease to baseline values at the end of the protocol (group A, 8.0 ± 1.5; group B, 8.4 ± 1.5; group C, 6.1 ± 0.9; *P* = 0.05).

In the mechanical substudy, AUC analysis of the induced decrease in cooled solution temperature revealed significant differences between the coolants. Addition of normal saline led to more intense cooling than colloid solution (−7155 ± 647 versus −5733 ± 636°C/minute, *P* = 0.008).

## Discussion

The main finding of the present study is that in this model a large volume of cold intravenous infusion of normal saline results in a more intense decrease in cerebral BT than colloid infusion. The method of intravenous cooling has been found efficient and safe. Infusion of 15 to 30 ml/kg of 4°C cold crystalloid at an infusion rate >60 ml/h may induce a decrease in BT >1.4°C [4-9]. However, there is significant inter-individual variability of cooling efficiency. Even administration of up to 30 ml/kg of cold fluid ensures the target therapeutic temperature of ≤34.0°C in fewer than 50% of patients [4,8,10]. There are several circumstances that may influence the cooling efficacy: inaccurate technique for BT measurement, under-dosing of the cold infusion, re-warming of coolant during its administration, type of intravenous access, cooling responsiveness of the patient and the type of coolant [10].

In theory, cooling efficacy of a coolant per se can be determined in two ways. First, there are different chemical and physical properties primarily expressed by different SHC of the coolant. Second, different chemical composition may influence thermoregulatory processes in the organism by affecting thermal exchange effectiveness, just as different fluids have a different impact on hemodynamics. In the presented pilot study, we focused on the comparison of cooling efficacy of both coolants, and we attempted to assess whether any potential difference may be affected by different SHC.

Vanden Hoek *et al*. compared the cooling effect of microparticulate saline slurry (MPS) versus chilled saline administered in 11 anesthetized farm swine with intact circulation [11]. MPS administration was associated with considerably greater decrease in cortical brain temperature than an equal amount of cold normal saline (5.3 ± 0.7 versus 3.4 ± 0.4°C, *P* = 0.009). MPS represents a potential solution designed for more efficient cooling. However, it is not manufactured and not available on the market. The authors also have presented an excellent model of heat content calculation for normal saline and MPS. Consistently with other results, it was higher for MPS than for normal saline. Although the model includes several approximations (specific heat of the body, virtual presumption of proportional distribution of the coolant in the body, ice load estimation in MPS), which may bring uncertainty into interpretation of the results, it represents a valid model for conducting further research.

Recently, Miclescu *et al*. have published an experimental study evaluating the cerebral and hemodynamic consequences of intravenous infusion of different volumes of cold Ringer’s solution or cold hypertonic hyperoncotic solution for induction of TH in a pig model of resuscitated cardiac arrest [12]. The animals were randomized to receive either 30 ml/kg administration of 4°C cold acetated Ringer’s solution (M group, n = 10) or 3 ml/kg of 4°C of hypertonic saline 7.5% - dextran 6% solution (L group, n = 10) during 30 minutes. Although administration of colloid solution was associated with greater reduction in cerebral edema than crystalloid infusion, the M group animals exhibited less neuronal damage than those in the L group. Thus, these observations remain inconclusive as to whether one of the treatments exhibits a clear neuroprotective effect. Even though the median time to target pulmonary artery temperature of 34°C was comparable in both groups (M group, 48.8 ± 8.6 minutes; L group, 51.5 ± 7.8 minutes; *P* >0.05), from the physical point of view this cannot be an expression of 10-fold higher cooling effectiveness of colloid compared to Ringer’s solution, and there is no doubt that additional external cooling via ice packs played a role.

In our experiment, a more intense decrease in cerebral and pulmonary artery BT at the time point of the end of the infusion was found in the group with normal saline, and the AUC for the cerebral BT decrease throughout the complete protocol indicated a more pronounced cooling effect of normal saline than of colloid solution. The question is what the major mechanism of this difference is.

SHC is the amount of heat per unit mass required to raise the temperature by one degree Celsius. In the clinical setting of TH induced by cold infusion, it means that the higher the SHC of the coolant, the more heat must be expended by the body to re-warm itself, which is an expression of more intense cooling efficacy. It is known that water has a high SHC of 4180 J/kg/°C, due to which it is used as an effective coolant in industry. Saline solution has a lower SHC, which decreases with the concentration of NaCl [13]. Blake *et al*. referred to the SHC of gelofusine as 4082 j/kg/°C [14]. Nevertheless, the SHC of novel colloid solutions has never been published. In our mechanical substudy, we did not measure absolute SHC values, but instead calculated the AUC of temperature changes for both coolants, a reflection of their relative SHC. As expected, we found this lower for colloid solution than for normal saline, and the difference appears clinically relevant. Thus, normal saline offers an advantage over an isotonic iso-oncotic solution of hydroxyethylamylum in normal saline in terms of physical and chemical properties directly related to cooling. Extrapolated to our animal experiment, it is thus reliable that the chemical composition of the investigated coolants is responsible for observed differences in cooling effect *in vivo*.

With respect to the impact of the investigated fluids on the process of thermal exchange within the body, it is not possible to draw any conclusions. We observed no relevant differences in hemodynamic variables throughout the protocol, and this will be the subject of further experiments. Other aspects to consider are whether intervention with different coolants may produce any adverse effects. After the cooling procedure, we did not observe any arrhythmia or signs of pulmonary edema. No unintentional cardiac arrest and no post-cardiac arrest shock syndrome developed in any animal after the ROSC. Massive volume-expansion in groups A and B had an impact on CPP and CoPP. Although in both groups we observed swifter restoration to the baseline values than in group C, colloid solution induced a distinct rise in ICP, which may be deleterious and disqualifying for clinical application. It is true that in our experiment we administered extreme doses of cold fluids at high infusion rate, supranormal to clinical dosage. However, significant ICP increase was already observed halfway through the intravenous cooling, which is equivalent to an application of 20 to 25 ml/kg. Expectedly, both colloid and crystalloid infusions induced an increase in the cardiac index [15].

There are some other limitations of our study, especially when considering its potential introduction into human medicine. We did not evaluate the impact of the intervention on neuronal damage, and we did not investigate whether the same amount of normal saline and colloid solution at room temperature has the same hemodynamic consequences as the cold solutions, performing the pilot study. The experiment was performed on animals without any evidence of heart disease before cardiac arrest, the dose of administered coolant was far beyond the therapeutic scope, and in general colloid infusion should be stored and used at room temperature.

However, we did not detect any apparent evidence of benefit of cold colloid infusion, and we should also be aware of known adverse events of colloid infusion, such as coagulopathy and the risk of renal injury in the clinical setting of post-cardiac arrest shock syndrome. Moreover, colloids are more expensive than crystalloids.

## Conclusions

In conclusion, large-volume cold intravenous infusion of normal saline resulted in more intense decrease in cerebral and pulmonary artery BT than colloid infusion of the same temperature and volume in this model of cardiac arrest. This difference is at least partially related to the various SHC of the coolants. Whether the different cooling effects of coolants may also be mediated through their different impact on thermoregulatory processes in the body is to be assessed in further experiments. Administration of colloid infusion was associated with temporary unacceptable increase in intracranial pressure. Considering other known adverse effects of high doses of colloids, we cannot recommend their regular use for induction of TH.

## Key messages

•Large-volume intravenous infusion of cold normal saline resulted in more intense decrease of cerebral and pulmonary artery body temperature than colloid infusion in this porcine model of cardiac arrest

•This difference is at least partially related to the various specific heat capacities of the coolants.

•Administration of colloid infusion was associated with temporary unacceptable increase of intracranial pressure.

•Considering the points above, we cannot recommend their regular use for induction of therapeutic hypothermia.

## Abbreviations

AUC: Area under the curve; BT: Body temperature; CoPP: Coronary perfusion pressure; CPP: Cerebral perfusion pressure; CVP: Central venous pressure; ECG: Electrocardiogram; FiO2: Inspired oxygen fraction; ICP: Intracranial pressure; MAP: Mean aortic pressure; MPS: Microparticulate saline slurry; ROSC: Return of spontaneous circulation; SHC: Specific heat capacity; TH: Therapeutic hypothermia; VF: Ventricular fibrillation.

## Competing interests

The authors declare that they have no competing interests.

## Authors’ contributions

All authors designed the study and prepared the protocol. RS and AT were the main investigators of the study. AT, ZT, RP, PD, SH, ChL and VC carried out experiments. All authors were involved in processing and interpretation of data. All authors drafted and revised the manuscript. All authors read and approved the final manuscript.
